# Digestion models: predicting the allergenic risk of novel food proteins

**DOI:** 10.1186/2045-7022-1-S1-S23

**Published:** 2011-08-12

**Authors:** Rod Herman

**Affiliations:** 1 Dow AgroSciences LLC, Biotechnology Regulatory Science, Indianapolis, IN, USA

## 

There is no definitive method to determine if a novel food protein will become an allergen. For this reason, the allergenic risk of a novel food protein is currently evaluated using a weight-of-evidence (WOE) approach. The components of this WOE approach were arrived at based on expert opinion rather than evidential science. Digestive stability is considered a physiochemical property of food allergens. For food allergens to interact with the immune system via the intestinal mucosa, they must survive the gastric digestion process in an immunoreactive state. An in-vitro assay based on simulated gastric fluid (SGF; 0.32% pepsin at pH 1.2) has been adapted for use with purified proteins to evaluate the stability of the primary structure of novel food proteins. SGF results are currently used as one component in a WOE approach for predicting the risk that a novel food protein will become an allergen. The SGF assay was adopted based an initial investigation that indicated several known allergens were more resistant to SGF compared with several non-allergens. Subsequent investigations have called this correlation into question. In addition, artifacts associated with interpretation of stability results using semi-quantitative approaches have been identified and more classical stability models have been used to enable quantitative comparisons among proteins; however, improved data interpretation has not supported a strong correlation between digestive stability in SGF and the allergenic status of food proteins. Potential interactions between proteins and food matrices have been identified as one factor that may contribute the poor predictive capability of this assay, as has the non-physiological conditions present in SGF. More physiological digestion models that include the food matrix have been suggested based on expert opinion, but as yet, no such assays have been shown to better discriminate food allergens from non-allergens compared with the SGF assay. More useful elements of the WOE approach include the source of the gene/protein, the prevalence of the protein in food, and the structural relationship with known allergens.

